# Scottish Matrons' Association

**Published:** 1921-04-02

**Authors:** 


### Scottish Matrons' Association.

The Annual Meeting of the Scottish Matrons' Association was held in Glasgow on March 19, Miss Gill, R.R.C., President of the Association, in the chair. There was a good attendance of members.

The Annual Report for the year 1920-21 was submitted and adopted. The honorary officers were re-elected; two members of the Council, Miss Filley and Miss Walker, retired; and Miss Davidson, R.R.C., and Miss Milligan, R.R.C., were elected to fill the vacancies. Miss Wise resigned the Hon. Treasurership after eight years of devoted work, and Miss Cumming, R.R.C., was appointed to succeed her. Five new members were enrolled.

The Chairman, in opening, paid a tribute to the memory of Miss Guy, R.R.C., late Lady Superintendent of the Royal Victoria Hospital, and one of the founder members of the Association. Miss Guy was a pioneer in her special line of work, and a noted authority on tuberculosis nursing.

Miss Melrose, R.R.C., reported the satisfactory progress of the scheme for the Home for Retired Nurses, which it is hoped shortly to open in Glasgow. The efforts of the various hospitals on behalf of the Home are proving most successful, and funds are coming in rapidly.

The Unemployment Insurance Act as it affects nurses was further discussed, the members present expressing themselves unanimously against the inclusion of nurses. It was resolved to send a Resolution on the subject to the Minister of Labour.

### Fever and Cottage Nurses and the Register.

Miss Gill, in speaking on the Registration of Nurses (Scotland), said that the General Nursing Council hoped soon to issue the Syllabus of Training. She explained two points on which a good deal of misunderstanding had arisen and some misrepresentation. The first, with regard to nurses holding the certificate of the Local Government Board of Scotland. Under the Act such nurses are qualified to go on the Register as existing nurses. The L.G.B. issues two certificates—namely, the certificate for their General Register, and that for their special Fever Register, and the claim had been made that nurses holding either certificate would be entitled to go on the general part of the State Register. The standard of the L.G.B. examination was well known in Scotland. Nurses holding the certificate must have had a three years' training, and been taught according to the Syllabus of the L.G.B. The number of nurses holding the L.G.B. fever certificates had been inquired into, and it was found that the majority had, subsequent to gaining their certificate, taken general training; as the examination had only been in existence for some nine or ten years, the number of fever nurses holding the L.G.B. certificate who had no subsequent qualification was so small as to be almost negligible among the numbers of existing nurses who would go on the Register. It was pointed out that no claim to go on the general part of the Register was made on behalf of any fever nurses excepting those who held the L.G.B. certificate after examination, and that this, of course, only applied to existing nurses (namely—prior to 1920). The other point on which misunderstanding had arisen was that of the cottage nurse. It had been stated in the Press that the General Nursing Council (Scotland) was acting on behalf of the cottage nurses. This was a mistake. The Scottish Council had asked the views of the English Council on the subject, a necessary proceeding, as there must be uniformity of standards. But the Scottish Council held no mandate for cottage nurses. For her own part, and she thought in this she voiced the views of the other nurse members of the Council, she would be very sorry to see cottage nurses admitted to the Nurses' Register.

